# Strontium and oxygen isotopes to trace mobility routes during the Bell Beaker period in the north of Spain

**DOI:** 10.1038/s41598-021-99002-8

**Published:** 2021-10-01

**Authors:** L. A. Ortega, C. Alonso-Fernández, I. Guede, M. C. Zuluaga, A. Alonso-Olazabal, J. Jiménez-Echevarría

**Affiliations:** 1 Department of Geology, Faculty of Science and Technology, University of the Basque Country-UPV/EHU, Sarriena s/n, 48940 Leioa, Bizkaia, Spain; 2 Cronos S.C. Arqueología y Patrimonio, C/ Aparicio y Ruiz 16-4, 09003 Burgos, Spain

**Keywords:** Biogeochemistry, Geochemistry

## Abstract

Strontium and oxygen isotopes of individuals from El Hundido and Valdescusa (north of Spain) sites, corresponding to the Bell Beaker culture, were analysed in order to determine mobility patterns and provenance areas. Strontium and oxygen isotope ratios in three teeth from two individuals at El Hundido and two teeth from the five individuals at Valdescusa were studied. The analyses were performed in both dentine and enamel fractions. ^87^Sr/^86^Sr ratios of El Hundido individuals indicate one was of foreign origin and the other was local whereas at Valdescusa were all of foreign provenance. Calculated δ^18^O_w_ values of El Hundido suggest a provenance from the geographical area close to the site while the Valdescusa would come from a warmer region. The comparison of oxygen and strontium isotope signatures indicate the west of the Iberian Peninsula (Zamora or the east of Leon regions) as the provenance area for the foreign individual at El Hundido and southwest France (Garonne basin) as the region of provenance for the Valdescusa.

## Introduction

The appearance of Bell Beaker pottery is a very important and controversial topic in archaeological research. After the discovery of the first Bell Beaker vessels in the Iberian Peninsula in the nineteenth century^1^ and the progressive appearance of similar ceramics and associated objects in most of Western Europe, during the twentieth century researchers searched for the Bell Beaker homeland and created models to explain the spread of the Bell Beaker culture. Schmidt^2^ proposed an Iberian origin for the Beaker "people" which was quickly adopted by Bosch Gimpera^3^. Del Castillo Yurrita proposed a "great Hispanic culture" that originated in the centre of the Iberian Peninsula and spread throughout Western and Central Europe, mainly motivated by the search for copper^4,5^. Del Castillo Yurrita, unlike Childe^6^, did not believe that the anthropological remains associated with Beaker finds corresponded to an ethnic unity, the Beaker folk.

Sangmeister^7^ proposed the "reflux model", which postulates a dual origin for the Bell Beakers. Initially, the Maritime Bell Beakers would emerge in the Tagus estuary area (Portugal) and spread in a "flow" movement through much of Western Europe. In Central Europe, this "culture" would hybridise with the local Chalcolithic ones in a "reflux" movement that would disperse them towards the South and West in advanced phases of the phenomenon, creating in each area different later local styles. However, the systematisation of radiocarbon dating soon led to this model being rejected.

In the 1970s a firmly constructed alternative hypothesis was advanced. The “Dutch model”, built first on typological arguments and later supported by radiocarbon dating, posited that Bell Beakers derived from Corded Ware in an area encompassing the Netherlands and the north-western part of Germany^8,9^. However, radiocarbon dating of Portuguese sites and the re-evaluation of sites in the Netherlands has led many experts to accept the Portuguese origin ^10^.

In the last decade of the twentieth century and the beginning of the twenty-first century, Bell Beaker research has been promoted on a European scale, based on anthropological and isotope mobility studies. Most scholar consider the Bell Beaker culture was a phenomenon based on the long-distance and fast-spreading movement of people, objects and ideas^11^. Studies in large Central European necropolises indicate that 50–60% of individuals correspond to migrants, regardless of gender or age^12^. A well-known case of long-distance mobility is the Amesbury archer^13^. In the Iberian Peninsula, the canonical Bell Beaker period is dated between 2500 and 2000 cal BC and characterised by simple tombs, often intrusive in earlier megalithic tombs, with a large repertoire of grave goods such as beakers and other vessels, Palmela points and personal adornments^14^. Late Bell Beaker or 'Epicampaniform' remains have been documented during the Middle Bronze Age in large regions of north-eastern Iberia^15^, southern France^16,17^ and in some regions of central Europe^18^ and are characterised by burials according to regional Bronze Age patterns.

Recent DNA studies enabled a better understanding of the complexity of the Bell Beaker period in the Iberian Peninsula and Europe. The study by Olalde et al.^19^ indicates an important contribution of steppe ancestry in the Iberian Beaker males (ca 2000 cal BCE). However, these authors detected limited genetic affinity between Beaker-complex-associated individuals in Iberia and central Europe, and thus exclude migration as an important mechanism of spread between these two regions. However, migration had a key role in the further dissemination of the Beaker complex.

This study does not attempt to discern whether Beaker folks or people with Beaker pottery exist. The aim of this work is focused on the contribution of isotopic geochemistry to determine the mobility of ancient individuals and establish movement patterns. In fact, the measurement of strontium and oxygen isotope ratios in teeth is considered a reliable method to assess the residential mobility and area of provenance of archaeological individuals due to the strong correlation between the isotopic composition of human tissues and the environment in which they lived.

## Archaeological background

**Figure 1:**
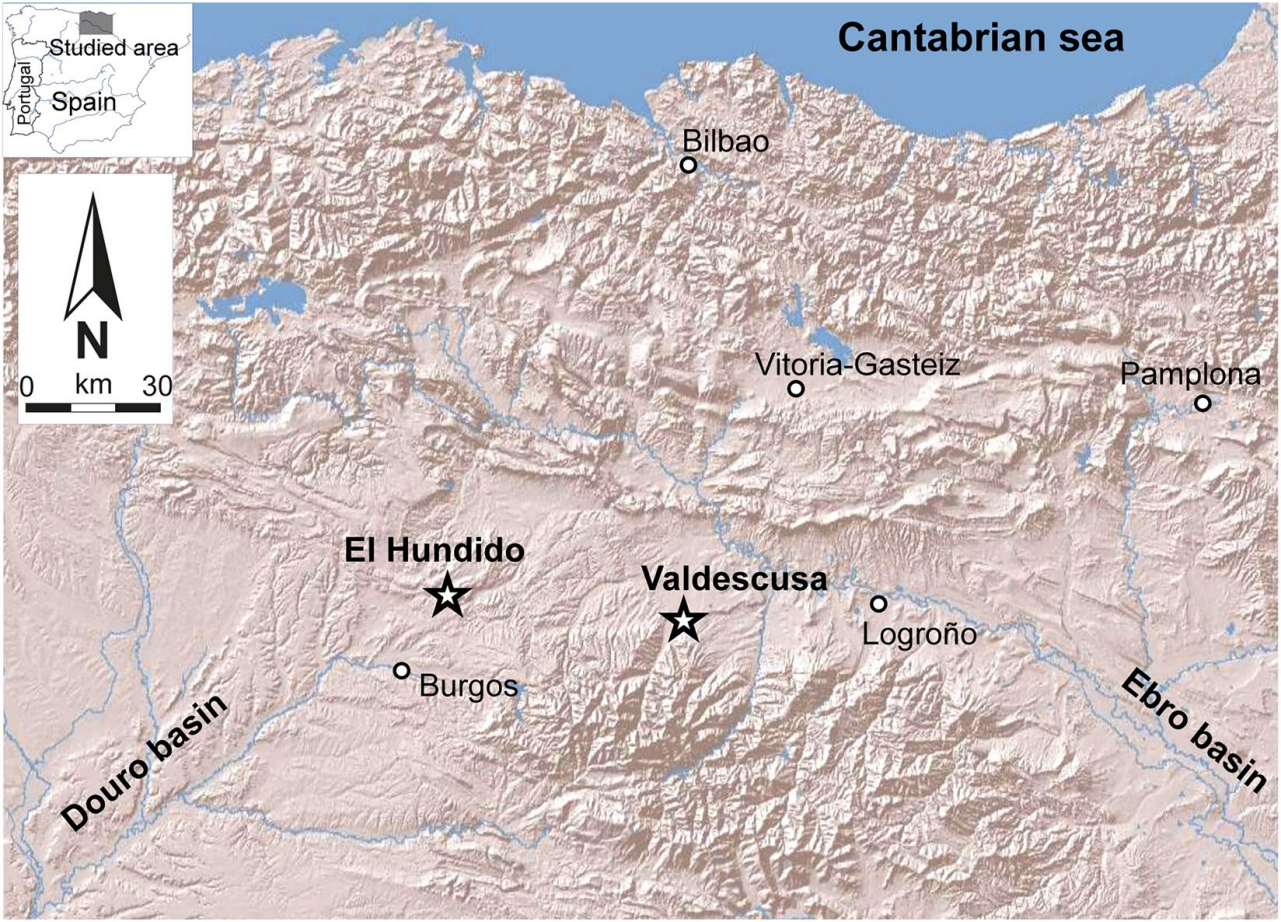
The geographical location of Valdescusa and El Hundido sites. Modified from Instituto Geológico y Minero de España (IGME). http://info.igme.es/visorweb/. Original copyright Geological Survey of Spain (IGME), under CC-BY license with permission from Instituto Geológico y Minero de España (IGME).

The studied sites correspond to two proximate locations on the interfluvial line between the Douro and Ebro river basins: El Hundido (Monasterio de Rodilla, Burgos) and Valdescusa (Hervías, La Rioja), respectively (Fig. 1).

The site of El Hundido is essentially a Chalcolithic collective burial where later, in the middle of the 3rd millennium BC, three Bell-Beaker intrusive burials were excavated^20^. However, in this study only samples of two individuals were analysed, since the alteration of Tomb 2 by looting caused the loss of most of the skeletal elements and prevented the recovery of any teeth. Burial UE450 corresponds to an adult male with an age at death of over 55 years old, dated to 2562–2306 cal BC, thus one of the oldest individuals of Bell Beaker people in this region. In the burial several grave goods were found consisting of a vessel with Ciempozuelos-style decoration, a copper Palmela point, a bone point and a pyrite sphere. The grave goods are characteristic of the Ebro Valley and show a clear trans-Pyrenean influence^20^. Burial UE750 corresponds to an adult male dated to 2287–2044 cal BC and did not have any associated grave goods (Table 1). The chronology dates the burials to the beginning of the Early Bronze Age on the Iberian Peninsula.

**Table 1:** List of samples for strontium and oxygen isotope analysis with main archaeological information.

Sample	Site	^14^C Age BP	^14^C Age Cal BC (2σ)	Sex	Age Group	Age (in years)	Tooth	^87^Sr/^86^Sr sample	δ^18^O sample
EH450-1	El Hundido	3933 ± 32	2490–2335	M	O A	> 55	26	Dentine, enamel	Enamel
EH450-3	El Hundido	3933 ± 32	2490–2335	M	O A	> 55	18	Dentine, enamel	Enamel
EH450-P	El Hundido	3933 ± 32	2490–2335	M	O A	> 55	35	Dentine, enamel	Enamel
EH750-1	El Hundido	3760 ± 30	2287–2044	M	A	50–55	16	Dentine, enamel	Enamel
EH750-2	El Hundido	3760 ± 30	2287–2044	M	A	50–55	37	Dentine, enamel	Enamel
EH750-C	El Hundido	3760 ± 30	2287–2044	M	A	50–55	33	Dentine, enamel	Enamel
Los Llanos	El Hundido							Water	
EH-S	El Hundido							Sediment	
E47	Valdescusa	3330 ± 30	1687–1517	M	I1	4 ± 1	84	Dentine, enamel	Enamel
E45M1	Valdescusa	3400 ± 35	1775–1609	M	A	25–35	16	Dentine, enamel	Enamel
E45M3	Valdescusa	3400 ± 35	1780–1610	M	A	25–35	48	Dentine, enamel	Enamel
E69	Valdescusa	3330 ± 30	1689–1528	M	I	6 ± 2	26	Dentine, enamel	Enamel
E74	Valdescusa	3479 ± 209 ^(^*^)^	1673–1255	M	J	12 ± 3	36	Dentine, enamel	Enamel
E77M1	Valdescusa	3360 ± 30	1700–1607	F	J	15 ± 3	16	Dentine, enamel	Enamel
E77M2	Valdescusa	3360 ± 30	1700–1607	F	J	15 ± 3	37	Dentine, enamel	Enamel
Cava del C	Valdescusa							Water	
VAL-S	Valdescusa							Sediment	

F: female, M: male, OA: old adult, A: adult, J: juvenile, I: infant.

*Thermoluminescence dating. Bayesian adjustment of the absolute chronology of the funeral pit E-45^28^.

The Valdescusa site was an open-air habitat with domestic and funerary structures characterised by stratigraphic and cultural synchrony between elements traditionally considered Bell Beakers and elements of the Middle Bronze Age. The funerary structures at the site include five individual burials in pits including two children (E47, E69), two young individuals (E74, E77) and one adult (E45) (Table 1). The funerary ritual differs from the canonical Bell Beaker burials, i.e. characterized by the lack of grave goods and scarce personal adornments. The pottery with Bell-Beaker motifs represents 10% of the total record in Valdescusa and corresponds to “Silos-style” ware, a variety of the Ciempozuelos-style similar to the pottery found in El Hundido, but with characteristics typical of La Rioja and the border of the Meseta. The chronology of the site obtained by the combined range of the five individuals is dated in 1685–1617 cal BC and corresponds to the end of the regional Bell-Beaker culture.

## Materials

The analysis of bones has been excluded since they are more susceptible to diagenetic alteration than teeth. Diagenetic susceptibility is linked to the size of the bioapatite crystallite. Bones have small crystallite size and high porosity and organic content, making them extremely susceptible to recrystallization and isotope alteration, even in compact bone^21^. Dentine has a similar crystallite size to bone but much lower porosity, reducing susceptibility to alteration. Enamel is extremely compact and has larger crystallites making it practically unsusceptible to diagenesis^22^.

Three teeth were analysed from each of the two individuals from the El Hundido site, including molars, premolars and canines. In the selection of the teeth to be analysed, the time of formation of the tooth was considered, limited by their availability^23^. In order to cover the largest time span of life of the individuals both enamel and dentine were analysed. After cleaning the surface, two fractions from each tooth were extracted with a dentist's microdrill (MF-Perfecta). The enamel sample was taken transversally to the tooth. Teeth of the five individuals at the Valdescusa site were analysed. From each individual, two molars were analysed; where possible, the first and the second or third maxillary or mandibular right molars (M1 and M2 or M3). The infant individual E47 had only deciduous dentition and one M1 molar was analysed (Table 1).

To determine the local bioavailable strontium isotope signature, samples of nearby sediments and water from streams near the archaeological sites were considered since they reflect the average isotopic composition of the catchment area covering a larger area, especially when the region is geologically homogeneous (i.e. the Ebro basin or the Douro basin). Fauna (archaeological or present-day, both wild and domestic) were discarded since may have a foreign character^24–27^. Besides, plants were also discarded since they record a strongly local signature. Runoff waters and sediments can determine the baseline at a regional scale and it is unnecessary to multiply the analysis of plant or soil samples.

## Results

The results of the strontium isotope analyses are summarized in Table 2 and the oxygen isotopes data in Table 3.

**Table 2:** Values of strontium isotopes ratios of enamel and dentine samples with standard error.

Sample	Site	Material	Tooth	^87^Sr/^86^Sr	1SE (abs)
EH450-1D	El Hundido	Dentine	26	0.709591	0.000008
EH450-1E	El Hundido	Enamel	26	0.712625	0.000010
EH450-3D	El Hundido	Dentine	18	0.709271	0.000008
EH450-3E	El Hundido	Enamel	18	0.709235	0.000006
EH450-PD	El Hundido	Dentine	35	0.709259	0.000006
EH450-PE	El Hundido	Enamel	35	0.712629	0.000009
EH750-1D	El Hundido	Dentine	16	0.70949	0.000007
EH750-1E	El Hundido	Enamel	16	0.709111	0.000007
EH750-2D	El Hundido	Dentine	37	0.709332	0.000008
EH750-2E	El Hundido	Enamel	37	0.709488	0.000008
EH750-CD	El Hundido	Dentine	33	0.709308	0.000007
EH750-CE	El Hundido	Enamel	33	0.709081	0.000008
Los Llanos	El Hundido	Water		0.709022	0.000010
EH-S	El Hundido	Sediment		0.70906	0.000008
E47D	Valdescusa	Dentine	84	0.71016	0.000004
E47E	Valdescusa	Enamel	84	0.710267	0.000009
E45M1D	Valdescusa	Dentine	16	0.71018	0.000004
E45M1E	Valdescusa	Enamel	16	0.709966	0.000004
E45M3D	Valdescusa	Dentine	48	0.710079	0.000004
E45M3E	Valdescusa	Enamel	48	0.709916	0.000004
E69D	Valdescusa	Dentine	26	0.710173	0.000005
E69E	Valdescusa	Enamel	26	0.710014	0.000004
E74D	Valdescusa	Dentine	46	0.709801	0.000003
E74E	Valdescusa	Enamel	46	0.709781	0.000005
E77M1D	Valdescusa	Dentine	16	0.709933	0.000003
E77M1E	Valdescusa	Enamel	16	0.71003	0.000004
E77M2D	Valdescusa	Dentine	37	0.709902	0.000004
E77M2E	Valdescusa	Enamel	37	0.709955	0.000005
Cava del C	Valdescusa	Water		0.708626	0.000004
VAL-S	Valdescusa	Sediment		0.708231	0.000005

**Table 3:** Stable oxygen isotope data reported as δ^18^O_p_ of the phosphate group of bioapatite and water.

Sample	Site	Material	Tooth	δ^18^O_p_ V-SMOW	± 1 σ, n = 3	δ^18^O_w_ I&V (2015)
EH450-1	El Hundido	Enamel	26	16.42	0.17	− 7.9
EH450-3	El Hundido	Enamel	18	15.87	0.21	− 8.9
EH450-P	El Hundido	Enamel	35	16.99	0.15	− 6.8
EH750-1	El Hundido	Enamel	16	16.2	0.2	− 8.3
EH750-2	El Hundido	Enamel	37	16.27	0.32	− 8.1
EH750-C	El Hundido	Enamel	33	16.26	0.25	− 8.2
E47E	Valdescusa	Enamel	84	17.66	0.07	− 5.6
E45M1E	Valdescusa	Enamel	16	17.35	0.01	− 6.2
E45M3E	Valdescusa	Enamel	48	17.18	0.05	− 6.5
E69E	Valdescusa	Enamel	26	18.1	0.09	− 4.8
E74E	Valdescusa	Enamel	46	18.28	0.5	− 4.4
E77M1E	Valdescusa	Enamel	16	17.78	0.12	− 5.4
E77M2E	Valdescusa	Enamel	37	17.22	0.06	− 6.4

### Evaluation of diagenesis

**Figure 2:**
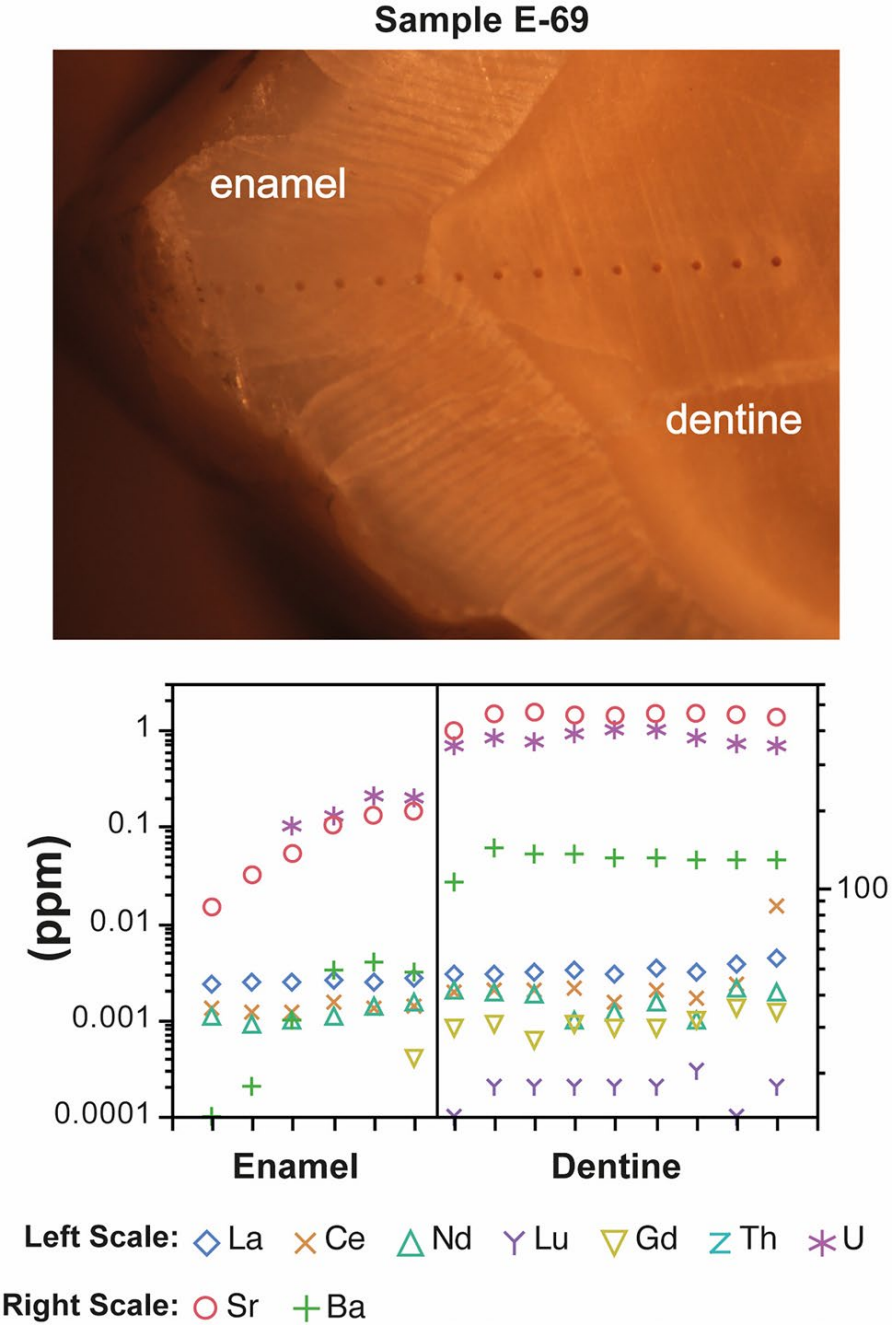
Top: Photomicrograph of the lower left second molar of Individual E69 from the Valdescusa site. The spots generated during the analysis by LA-ICP-MS can be observed (original fotograph by LA Ortega). Bottom: content (in ppm) of elements sensitive to diagenesis.

To guarantee that isotope ratios measured in tooth reflect the original isotopic composition, it is necessary to ensure the lack of diagenetic alteration^29^. In archaeological samples, burial conditions can cause changes in the mineral composition leading to exchange of ions with the environment^30^. Several methods have been proposed to assess diagenesis in teeth and bones, such as the degree of collagen preservation, crystallinity index, Ca/P ratio, etc.^31,32^. However, the application of these methods has been fiercely debated as they do not provide information on the degree of chemical modification in bones^33,34^. In contrast, rare earth elements (REE) and uranium (U) are considered highly sensitive markers of diagenetic modification. During burial, U and REE replace very effectively the hydroxyl group (OH-) and Ca of the bioapatite, thus tracing the possible alteration and modification of other chemical elements^35–39^. U, Th and REE contents in the teeth were analysed by laser ablation (LA-ICP-MS) to determine the degree of preservation. Most of the samples show similar low contents to those determined by Kohn et al.^40^ in present-day herbivores, carnivores or omnivores (typically < 0.001 ppm) proving the absence of diagenesis. Only the dentine fraction of sample E69 (corresponding to a child about 6 years old) shows U contents close to 1 ppm suggesting very incipient diagenesis (Fig. 2). Strontium isotope composition does not seem to be modified since the Sr content pattern does not show spatial variations.

### Strontium isotope ratios

**Figure 3:**
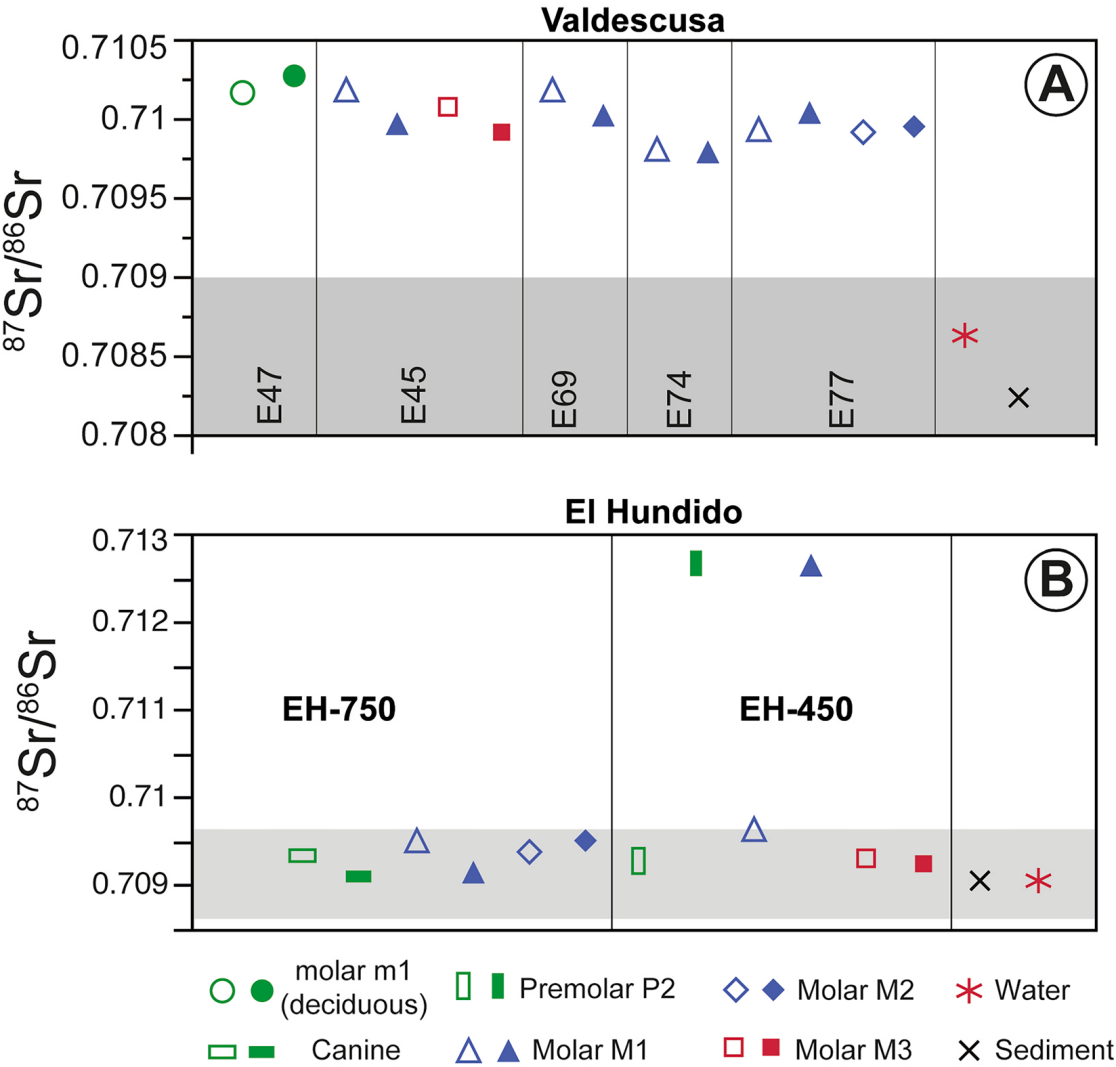
Strontium isotope ratios of tooth enamel and dentine, water and sediments close to the sites. The grey area corresponds to the local bioavailable strontium isotope signature range calculated as ± 1.5 IQR (interquartile range) of the median of sediments and water values.

The ^87^Sr/^86^Sr ratios of the teeth of the El Hundido individuals range from 0.70908 to 0.71263. In general, these values are similar to the ^87^Sr/^86^Sr ratios of the sediments and runoff water ranging between 0.70902–0.70906 (Table 2, Fig. 3a). These values are in agreement with the isotope values at the Alto de Reinoso site, located very close to El Hundido and on the same geological materials. The grey area in Fig. 3 indicates the regional strontium isotope baseline defined as ± 1.5 IQR (interquartile range) of the sediment and water values including data from Alto de Reionoso and El Prado sites^41^. However, the Sr isotopic composition of the enamel in premolar P1 and molar M1 of Individual E450 reflects values highly enriched in radiogenic strontium, with ^87^Sr/^86^Sr ratios = 0.7126. These values indicate that Individual E450 lived in a geological setting far from the site during the enamel mineralization of these teeth.

The ^87^Sr/^86^Sr ratios of the teeth of individuals from the Valdescusa site range from 0.70978 to 0.71027 showing higher values than the sediments and the local fresh water, with ^87^Sr/^86^Sr = 0.70823 and ^87^Sr/^86^Sr = 0.70863, respectively (Table 3, Fig. 3b). The teeth of each individual do not show significant variations in isotopic composition, either between the enamel and dentine of the same piece, or between different teeth of the same individual. Only sample E45 corresponding to an adult male shows a small difference of over 0.0002 in the ^87^Sr/^86^Sr ratio between enamel M1 and dentine M3 (Fig. 3). Even considering all the individuals, scarce variation in the isotopic composition is observed with Δ^87^Sr/^86^Sr = 0.0005. The isotope composition of the individuals’ distance from the local isotope range is defined as ± 1.5 IQR (interquartile range) of the sediment and water values (Fig. 3) indicating their foreign provenance.

### Oxygen isotope composition

Table 3 shows oxygen isotope values of the phosphate anion (δ^18^O_p_) of enamel subsamples and the conversion to drinking water isotope values (δ^18^O_w_) using the equation in Iacumin and Venturelli^42^. Enamel phosphate oxygen ratios of human individuals from El Hundido site showed that δ^18^O_p_ values range between 15.87 and 16.99‰ with an average value of δ^18^O_p_ = 16.34 ± 0.37 (1σ)‰. In contrast, δ^18^O_p_ values of human individuals from the Valdescusa site show slightly heavier values ranging from 17.2 to 18.3 ‰ with an average value of δ^18^O_p_ = 17.7 ± 0.47 (1σ)‰.

The δ^18^O_w_ calculated for teeth of individuals from El Hundido ranges from − 8.9 to − 6.8‰, with a mean value of − 8.0‰ ± 0.7‰, (1σ) while the δ^18^O_w_ for teeth of individuals from Valdescusa ranges from − 6.5 to − 4.4‰ with a mean value of − 5.6‰ ± 0.8‰ (1σ).

## Discussion

Since each isotopic system serves as a proxy of the geological (in the case of strontium) or geographical and/or environmental (case of oxygen) setting, the strontium and oxygen data will first be discussed separately and then in combination.

The studied sites are located on upper Miocene continental sediments consisting mainly of conglomerates, sandstones, shales with limestones, marls and occasionally gypsum (Supplementary Fig. S1). Although the materials in the geological environment of the sites are similar in composition and age, the isotope baseline for bioavailable strontium is slightly different with values of ^87^Sr/^86^Sr = 0.7090 for El Hundido and ^87^Sr/^86^Sr = 0.7084 for Valdescusa (Fig. 3).

According to the strontium local baseline, the variation in the isotopic composition of the individuals from each site indicates a foreign origin of most of them, but with different characteristics. Individuals from El Hundido show values of ^87^Sr/^86^Sr in the range of local sediments and waters in all the analysed dentine fractions. However, enamel samples of Individual EH450 show a wide variation in the strontium isotopic composition indicating a different geological environment at the time of mineralisation of the tooth. The most recent individual (EH750) displays a similar isotopic composition to the local composition for both dentine and enamel. These results indicate that Individual EH750 lived in the area of the site throughout his life from childhood to maturity without any residence change. However, the oldest individual EH450 shows significant enrichment in radiogenic strontium, with ratios of ^87^Sr/^86^Sr = 0.7126 in the enamel of the premolar P1 and molar M1 suggesting a foreign origin. Such high values in the ^87^Sr/^86^Sr ratio are typical of granitic sediments common in the geology of the western Iberian Peninsula, but also in some granitic environments of the Pyrenees, pointing to those potential provenance areas for the latter individual.

According to Knipper, the enamel of the M1 molar forms between the last month of pregnancy and two and a half years of age, while dentine may form as late as ten years of age. Enamel mineralisation of the second premolar P2 can last from the second year of life to six years, while dentine can last up to 12 years. The M3 third molars are the only teeth that form after the weaning period and correspond to the most variable teeth in terms of their development, with enamel forming between 7 and 13 years of age while dentine mineralisation may last up to 20 years^43,44^.

Considering the periods in the formation of the dentition and the variation in the isotopic composition of the El Hundido individuals, it can be proposed that Individual EH450 would have been born in a geological environment away from the site, possibly in the granitic areas in the western part of the Iberian Peninsula. This male EH450 would remain in this geological setting until at least two years of age and at most until 6 years of age, as indicated by the latest time of enamel formation of the P2 premolar. Then, at an age of about 6 years, this individual would move to the El Hundido site, as indicated by the composition of the dentine of the different teeth, and would have settled in the area before the age of 8 years, the time corresponding to the formation to the third molar (M3) enamel.

The strontium isotopic ratios of individuals from the Valdescusa site vary very little, indicating not only a foreign origin of all individuals but also a geologically similar provenance area. The remarkable similarity in the ^87^Sr/^86^Sr ratio of the teeth of all individuals, both adults and infants, suggests either a long-time occupation in the same geological region with restricted mobility or mobility over a broad geologically similar region. Moreover, considering the time span in the chronology of the individuals (1685–1617 cal BC), the strontium isotopic ratios indicate an invariable mobility route of these individuals or populations over at least two or more generations.

The lack of regional maps of strontium isotope composition makes it difficult to propose provenance areas for these individuals. Supplementary Figure S1 shows the isotope composition of the Valdescusa site and other nearby archaeological sites (Neolithic or more recent). The strontium isotope baseline of the regional sites is different from that of the individuals studied here, excluding these regions as a provenance area and pointing towards a more remote region of origin.

The calculated δ^18^O_w_ values or isotopic composition for meteoric water or local intake of the individuals at El Hundido show a very small variation. It is less than the uncertainty associated with the calculation of this parameter, which is estimated at 2.5‰^42^. No differences in δ^18^O_w_ composition are observed between the local and the foreign individual. Only the canine tooth of Individual E750 shows more enriched δ^18^O_w_ values that could be explained as a breastfeeding effect, since enamel of canine teeth mineralises between the first and fourth year of life. In fact, it is well known that δ^18^O of human milk is enriched of about 1–2‰ over drinking water^45,46^.

δ^18^O_w_ values of individuals from Valdescusa measured in the M1 tooth (mineralised during the breastfeeding period) show variable and more enriched values than the δ^18^O_w_ measured in M3 tooth (formed after infancy). The variability in values of the M1 and M2 teeth may be due to their formation in different periods during weaning^46^.

**Figure 4:**
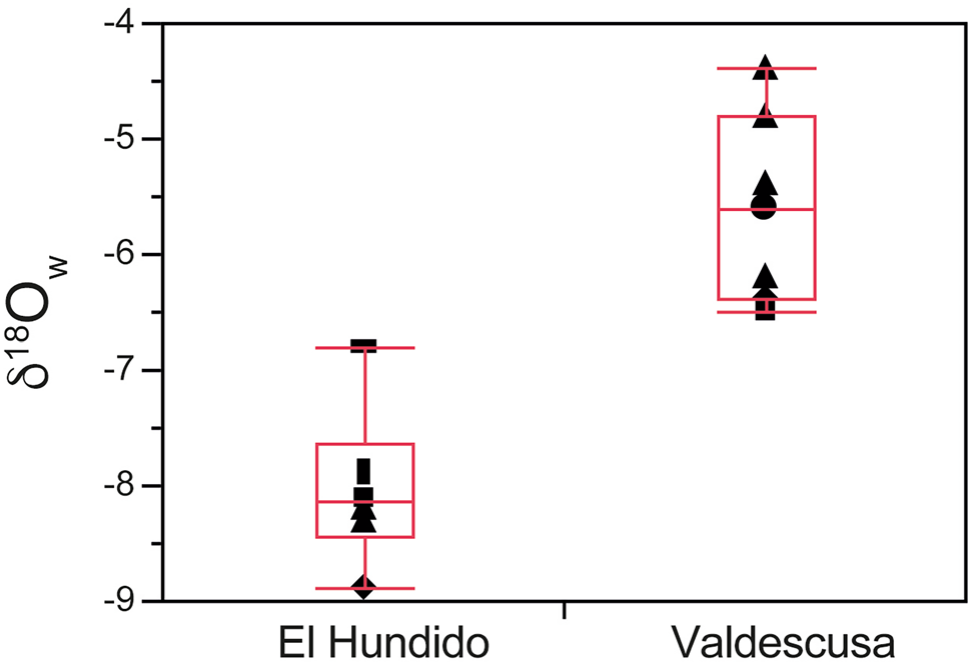
δ^18^O_w_ values recorded in tooth enamel of individuals from El Hundido and Valdescusa sites.

Although El Hundido and Valdescusa are located 50 km apart, they are in the same climatic zone (Cfb class) according to the Köppen classification. The meteorological information available from AEMET (Spanish Meteorological Agency) indicates both sites have experienced very similar temperatures, precipitation and hours of sunshine over the last 20 years. Data from meteorological stations at Santo Domingo de la Calzada, 6 km from the Valdescusa site, and Burgos-Villafría airport and Briviesca, 16 km SW and 17 km NE of El Hundido, respectively, have been considered. The average temperature in Monasterio de Rodilla (municipality of El Hundido site) is 1º C lower than in Santo Domingo de la Calzada, but the thermal amplitude is higher in the latter. Therefore, it should be expected that the intake water should have similar oxygen isotope composition at the two sites. However, when comparing the isotope composition of the two sites recorded in tooth enamel, it can be observed that the water ingested did not have similar isotopic composition (Fig. 4) (t-test: t = 5.94, gl = 11, p < 0.001; Mann–Whitney test; U = 42, p = 0.001). In order to understand these dissimilarities, δ^18^O_w_ values of the individuals of El Hundido and Valdescusa have been compared with expected local water values according to the IAEA/WISER database (https://nucleus.iaea.org/wiser/index.aspx). The individuals from El Hundido could come from the geographical area close to the site with δ^18^O_w_ values close to -8‰, while the individuals from Valdescusa should come from a region with heavier δ^18^O_w_ (− 6.5 to − 4.5‰), related to drinking water from slightly warmer regions.

The results of oxygen and strontium isotope composition indicate a foreign provenance of most of the studied individuals. The comparison of oxygen and strontium isotope signatures provide geographic and geological information that, combined with the information acquired over the years by numerous researchers, allows us to hypothesise about the regions of provenance of these Bell Beaker individuals. Table 4 includes the data available in the GNIP (Global Network of Isotopes in Precipitation) and REVIP (Red de Vigilancia de Isótopos en Precipitación, Spanish Government) databases for stations close to the sites and for other more distant areas that could be considered potential provenance areas for individuals of this culture.

**Table 4:** δ^18^O average values of meteoric water from different stations in the north and northwestern of Iberian Peninsula.

Site	GNIP			REVIP	
	Mean	SD	Years	Mean	Years
Burgos/Villafría	− 7.74	3.14	2002–2003		
Bordeaux (Cestas-Pierroton)	− 5.89	0.85	2007–2014		
Bragança	− 6.7	2.3	1988–1991		
Dax	− 4.91	2	1999–2004		
León/Virgen del Camino	− 7.65	3.4	2000–2010	− 8.44	2000–2015
Madrid-Retiro	− 6.3	3.35	1970–2010	− 7.2	2000–2015
Salamanca/Matacán	− 7.78	1.94	2002–2003		
Santander	− 5.19	1.78	2000–2010	− 5.72	2000–2015
Soria	− 7.86	4.32	2002–2003		
Valladolid	− 6.7	3.44	2000–2010	− 7.92	2000–2015
Zaragoza Aeropuerto	− 5.58	3.17	2000–2010	− 6.31	2000–2015
Toulouse (Université)	− 6.64	1.75	2011–2012		

The δ^18^O_w_ values for the individuals from El Hundido are similar to those of the stations in the Northern Meseta (Burgos, León, Salamanca, Ávila) and Northern Portugal (Bragança), suggesting this region is the most probable provenance area of the individuals from this site. The oldest individual (E450) with teeth formed during early childhood recording ^87^Sr/^86^Sr = 0.712 values must have come from Zamora or the east of Leon region, since the regional geological materials have very similar strontium isotope values^47,48^. Besides, the most recent individual (E750) shows isotopic signatures suggesting a local origin, but the migrant character of the Bell Baker population should not be ruled out. This individual would have been similarly mobile but through a geologically more homogeneous region. The possible area of mobility is limited to the Douro basin, which geologically shows remarkable homogeneity in the strontium content^49^. Besides, the Douro basin shows a suitable and homogeneous oxygen isotope composition^50,51^ and also strontium isotopic composition, as indicated by the scarce isotopic studies of the region^41,52^.

The δ^18^O_w_ values for the Valdescusa individuals are close to the values mapped by Capilla et al. for the surroundings of the site and similar to the middle Ebro basin (Zaragoza, Table 4). However, the strontium isotope composition rules out the Ebro basin^53,54^ and Cantabrian coastal region ^55,56^ as provenance areas. In contrast, the δ^18^O_w_ values point to south-western France (Cestas-Pierroton, Toulouse) as the provenance area. Besides, considering the IRHUM database^57,58^ for France, the strontium isotope composition of the Valdescusa individuals equally points to south-western France. Small variations in the strontium isotopic signature of teeth may be related to settlements in various locations within the Garonne basin, as is evidenced by small variations in the water composition of the different tributaries of the Garonne (see Fig. 1 in Semhi et al.^59^).

The isotopic study shows that the Bell Beaker individuals were mobile over long distances although the mobility patterns varied in different time periods. In the earliest times (El Hundido site) the mobility was within the Iberian Peninsula whereas in the Late Bell Beaker period (Valdescusa site) the individuals originated in the European continent.

## Analytical methods

This study deals with archaeological skeletal material and all necessary permits were obtained for the described study, which complied with all relevant regulations for the treatment of ancient human remains. The excavation licenses were issued by the General Directions of Cultural Heritage of the La Rioja and, Castile and Leon Governments (Spain) and are stored in its archives. Following excavation seasons 2010 and 2013, the bones and teeth samples with their permission were transferred to the Heritage and Cultural Landscape Research Group (GIPyPAC) at the University of Basque Country-UPV/EHU, Spain for investigation.

### Strontium isotope ratio (^87^Sr/^86^Sr)

For isotopic analysis of the teeth, about 10 mg of both enamel and dentine were weighed and dissolved in 7 mL Teflon vials (Savillex, Minnetonka, MN, USA) with 1.5 mL of 2 M HNO_3_ (analytical grade purified by surface evaporation distillation).

Freshwater samples were filtered through a 0.45 um filter to remove suspended particles and then 15 mL were taken and evaporated to dryness and then dissolved in 1.5 mL of 7 M HNO_3_. For sediment samples, 1 g was weighed and leached using 2.5 mL of 1 M ammonium nitrate (NH_4_NO_3_) and shaken for 8 h at 200 rpm to obtain the bioavailable Sr. Subsequently, the samples were centrifuged at 3000 rpm for 15 min, the supernatant liquid (~ 1–1.5 mL) was taken and brought to dryness and then redissolved in 1.5 mL of 2 M HNO_3_. All solutions were loaded onto cation exchange columns loaded with Sr-resin (Triskem International, Bruz, France), a strontium selective resin. The resin was used once to elute the sample and then discarded. The strontium process blank is less than 100 pg and therefore provides a negligible contribution.

The isotope ratios were measured in a mass spectrometer with a Neptune® multi-collector plasma source (MC-ICP-MS) in the Advanced Research Facilities (SGIker) at the University of the Basque Country-UPV/EHU. The measurement of the reference material NBS-987 indicates an external precision for the analyses of ± 0.00002 (2σ absolute), while the analysis of NBS 987 during the analysis period gives values of ^87^Sr/^86^Sr 0.710286 ± 10 (2σ, n = 8).

### Stable oxygen isotopes analysis

Silver phosphates were obtained following a modification of the methodology described by Dettman et al.^60^. Between 10 and 20 mg of either enamel or dentine sample were used. Organic matter was removed by reacting the sample with 1 mL of a 2.5% NaOCl solution for 24 h at room temperature. The sample was then reacted for 48 h in 1 mL of 0.125 M NaOH solution at room temperature. Following the removal of organic matter, the sample was reacted with 1 mL of 2 M HF for 36 h. The phosphate solution and the CaF2 compound residue were separated by centrifugation. The solution is pipetted into a 10 mL polypropylene tube, and the residue is washed three times with 1 mL ultrapure water. The solution is then neutralised with 0.8 mL of 6% NH_4_OH. Finally, the silver phosphate is precipitated by adding 2 mL of AgNO_3_. The silver phosphate is washed twice with ultrapure water, and the residue is filtered and dried in an oven at 50 °C.

For spectrometric measurements, 0.3 mg of Ag_3_PO_4_ was mixed with 0.5–1 mg of AgCl and 0.3 mg of graphite in tin capsules. The capsules were transferred to the Elemental Analyzer Autosampler (TCEA) carousel and degassed for 30 min at 80°C under vacuum. Oxygen isotope analyses were carried out on a Thermo-Finnigan TCEA elemental analyser coupled to a Delta Plus XP spectrometer at the University of Salamanca. The isotopic composition is expressed according to the conventional δ notation with respect to V-SMOW (Viennacx Standard Middle Ocean Water). The standardization of the V-SMOW scale was based on four replicated international reference materials provided by the International Atomic Energy Agency (IAEA): IAEA-601, IAEA-602, IAEA-CH6 and IAEA-SO-6. The analyses correspond to an average of three measurements from each sample and the analytical precision of each single determination is better than ± 0.3 ‰. In order to relate the δ^18^O_ph_ values of the PO_3_^–4^ anionic group of enamel to the δ^18^O_w_ of drinking water, the following equations were used:

- Iacumin y Venturelli^42^: δ^18^O_w_ = 1.847 × δ^18^O_p _− 38.40.

Statistical analyses were performed using PAST and SPSS licensed to the University of the Basque Country.

### Laser ablation-inductively coupled plasma-mass spectrometry (LA-ICP-MS)

Chemical analyses were carried out using a Thermo X7 quadrupole-inductively coupled plasma-mass spectrometer (Q-ICP-MS) updated to XSeries2, coupled to the UP213 laser ablation system equipped with Xt interface unit in the Advanced Research Facilities (SGiker) at the University of the Basque Country-UPV/EHU. Laser ablation system was New Wave Nd:YAG operating at a wavelength of 213 nm. Calibration of LA-ICP-MS was achieved using a standard reference glass material NIST SRM 612^61–63^. Measurements were taken for the spot size of 100 μm at a distance of 300 μm from each other. Time delay between the end of LA of one spot and the initiation of LA of the next spot was 15 s. LA was performed with a laser spot diameter of 100 μm, laser fluency 4.5 J/cm, and a repetition rate of 10 Hz. Data collection was performed by rapid peak-hopping (5–30 ms) dwell time between selected isotopes of each analyte element for a period of 60 s. A background signal was collected during the first 30 s of analysis. The laser was fired for 60 s from which the middle 30 s were used for signal integration. For data reduction, Iolite 3 data processing software package for time resolved mass spectrometry data was used^64^. The ^43^Ca was used as an internal standard to account for variation in ablation efficiency caused by variations in the mass of the material ablated. Calcium concentration was assumed from the stoichiometry of biogenic hydroxyapatite. The estimated calcium content is 252 mg/g for the dentine and 360 mg/g for the enamel tissues^35^.

## Supplementary Information

Supplementary Information.
