# Supplementary material for: Strontium and oxygen isotopes to trace mobility routes during the Bell Beaker period in the north of Spain
Source: Sci Rep. 2021 Oct 1;11:19553. doi: 10.1038/s41598-021-99002-8 (PMC8486826; doi:10.1038/s41598-021-99002-8)
Supplement: Supplementary file 1 — Supplementary Information. [file 41598_2021_99002_MOESM1_ESM.pdf]

# Supplementary Information

## Strontium and oxygen isotopes to trace mobility routes during the Bell Beaker period in the north of Spain.

L.A. Ortega*[1], C. Alonso-Fernández[2], I. Guede[1], M.C. Zuluaga[1], A. Alonso-Olazabal[1], J. Jiménez-Echevarría[2].

1 Department of Geology, Faculty of Science and Technology, University of the Basque Country-UPV/EHU, Sarriena s/n, 48940 Leioa, Bizkaia, Spain; luis.ortega@ehu.eus (L.A.O.), iranzulaura.guede@ehu.eus (I.G.), mcruz.zuluaga@ehu.eus (M.C.Z.), ainhoa.alonso@ehu.eus (A.A.-O.)
2 Cronos S.C. Arqueología y Patrimonio, C/ Aparicio y Ruiz 16-4 D 09003, Burgos, Spain; ca@cronossc.es (C.A.-F.), jj@cronossc.es (J.J.-E.)

**Corresponding author:**
Luis Ortega
Department of Geology, Faculty of Science and Technology,
University of the Basque Country UPV/EHU,
Sarriena s/n, 48940 Leioa, Spain.
e-mail: luis.ortega@ehu.eus

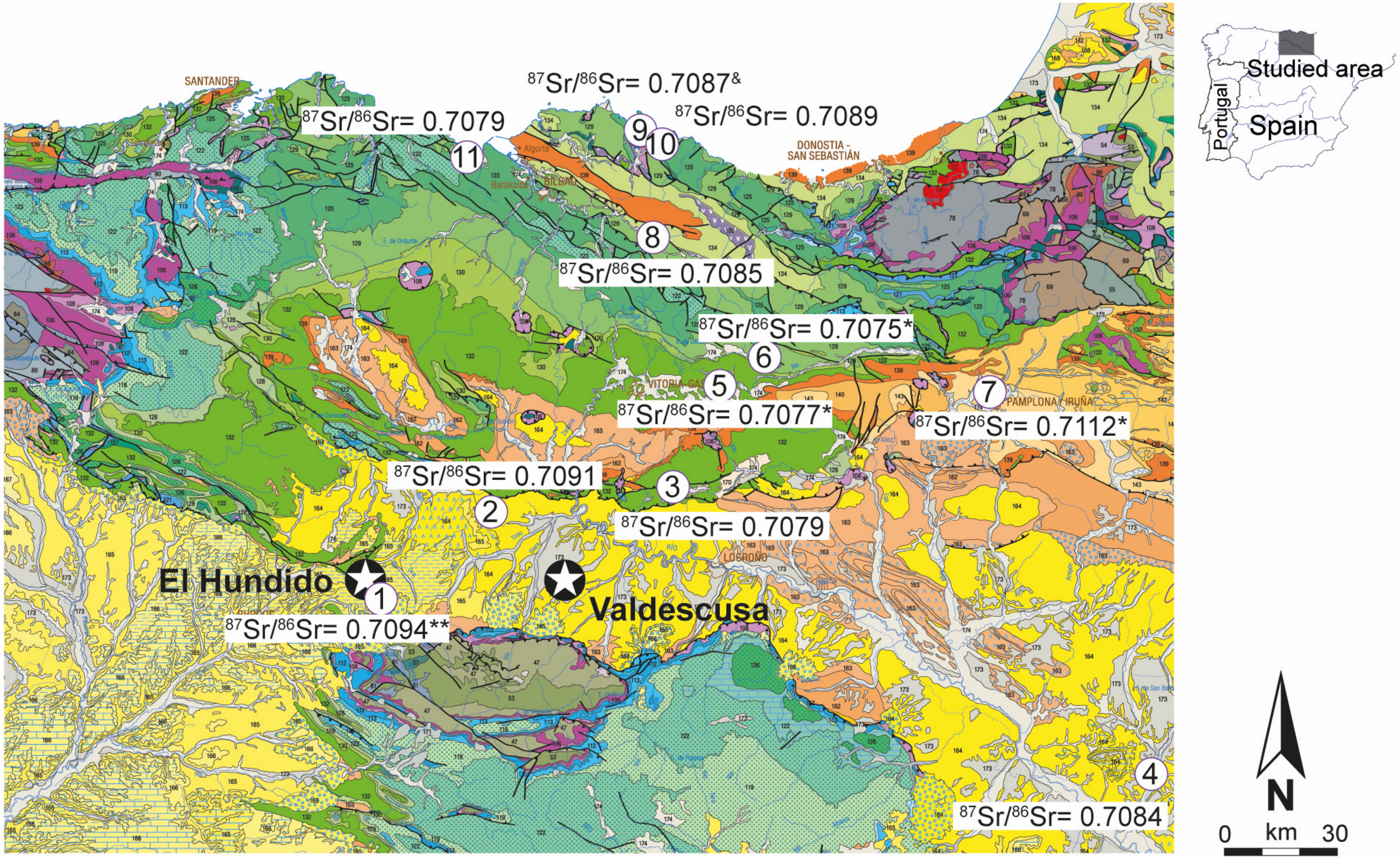


1: Alto de Reinoso[1], 2: El Prado[2], 3: Las Gobas[3], 4: Tauste[4], 5: Alegría-Dulantzi[5], 6: Aistra[6], 7: El Castillo[7], 8: Momoitio[8], 9: Santimamiñe[9], 10: Lumentxa[10], 11:Pico Ramos[9]

**Fig. S1**: Geological map with values of the strontium isotope baseline of regional archaeological sites estimated from runoff water. Except (*) from soil, (**) from archaeological fauna, (&) from plants. Reprinted from Rodríguez Fernández[11] under CC by license, with permission from Instituto Geológico y Minero de España (IGME), original copyright Geological Survey of Spain (IGME).

## References


1 Alt, K. W. *et al.* A community in life and death: The late neolithic megalithic tomb at Alto de Reinoso (Burgos, Spain). *PloS one* **11**, doi:10.1371/journal.pone.0146176 (2016).

2 Ortega, L. A., Guede, I. L., Zuluaga, M. C. & Alonso-Olazabal, A. in *Vida y muerte en el asentamiento del Neolítico Antiguo de El Prado (Pancorbo, Burgos). Construyendo el Neolítico en la Península Ibérica* Vol. S2876 (ed Carmen Alonso-Ferández) Ch. 10, 218 (BAR Publishing, 2017).

3 Guede, I. *et al.* Isotopic evidence for the reconstruction of diet and mobility during village formation in the Early Middle Ages: Las Gobas (Burgos, northern Spain). *Archaeological and Anthropological Sciences* **10**, 2047-2058, doi:10.1007/s12520-017-0510-9 (2018).

4 Guede, I. L. *et al.* Isotope analyses to explore diet and mobility in a medieval Muslim population at Tauste (NE Spain). *PloS one* **12**, e0176572, doi:10.1371/journal.pone.0176572 (2017).

5 Ortega, L. A. *et al.* Strontium isotopes of human remains from the San Martín de Dulantzi graveyard (Alegría-Dulantzi, Álava) and population mobility in the Early Middle Ages. *Quat. Int.* **303**, 54-63, doi:10.1016/j.quaint.2013.02.008 (2013).

6 Guede, I. *Movilidad de poblaciones en época alto-medieval en el norte peninsular a partir de la composición isotópica del Sr en dientes. Caso del yacimiento arqueológico de Aistra (Zalduondo, Alava)* Master thesis, Universidad del País Vasco-UPV/EHU, (2013).

7 Prevedorou, E. *et al.* Residential Mobility and Dental Decoration in Early Medieval Spain: Results from the Eighth Century Site of Plaza del Castillo, Pamplona. *Dental Anthropology* **23**, 42.52 (2010).

8 Guede, I. *et al.* Social structuration in medieval rural society based on stable isotope analysis of dietary habits and mobility patterns: San Juan de Momoitio (Biscay, north Iberian Peninsula). *Journal of Archaeological Science: Reports* **31**, doi:10.1016/j.jasrep.2020.102300 (2020).

9 Sarasketa-Gartzia, I., Villalba-Mouco, V., le Roux, P., Arrizabalaga, Á. & Salazar-García, D. C. Late Neolithic-Chalcolithic socio-economical dynamics in Northern Iberia. A multi-isotope study on diet and provenance from Santimamiñe and Pico Ramos archaeological sites (Basque Country, Spain). *Quat. Int.* **481**, 14-27, doi:10.1016/j.quaint.2017.05.049 (2018).

10 Guede, I. *Geochemical studies of palaeodiet and mobility of medieval communities of North Iberian Peninsula* doctorate thesis, Universidad del País Vasco-UPV/EHU, (2018).

11 Rodríguez Fernández, L. R. *et al.* (eds L. Roberto Rodríguez Fernádez & J. Tomás Oliverira) (Instituto Geológico y Minero de España & Laboratório Nacional de Energia e Geologia, I.P., Madrid, 2015).
